# Neurofilament light chain as a diagnostic and prognostic biomarker in Guillain–Barré syndrome

**DOI:** 10.1007/s00415-024-12679-5

**Published:** 2024-09-09

**Authors:** Brynhildur Hafsteinsdóttir, Helen Farman, Nina Lagerström, Henrik Zetterberg, Oluf Andersen, Lenka Novakova, Bengt Nellgård, Hans Rosén, Clas Malmeström, Igal Rosenstein, Jan Lycke, Markus Axelsson

**Affiliations:** 1 Department of Clinical Neuroscience, Institute of Neuroscience and Physiology, The Sahlgrenska Academy, University of Gothenburg, Gothenburg, Sweden; 2 Department of Neurology, Sahlgrenska University Hospital, Region Västra Götaland, Gothenburg, Sweden; 3 Department of Psychiatry and Neurochemistry, Institute of Neuroscience and Physiology, The Sahlgrenska Academy, University of Gothenburg, Gothenburg, Sweden; 4 Clinical Neurochemistry Laboratory, Sahlgrenska University Hospital, Mölndal, Sweden; 5 Department of Neurodegenerative Disease, UCL Institute of Neurology, Queen Square, London, UK; 6 UK Dementia Research Institute at UCL, London, UK; 7 Hong Kong Center for Neurodegenerative Diseases, Clear Water Bay, Hong Kong, China; 8 Wisconsin Alzheimer’s Disease Research Center, School of Medicine and Public Health, University of Wisconsin, University of Wisconsin-Madison, Madison, WI USA; 9 Department of Anesthesiology and Intensive Care, Sahlgrenska University Hospital, Mölndal, Sweden; 10 Department of Anesthesiology and Intensive Care, Institute of Clinical Sciences, Sahlgrenska Academy, University of Gothenburg, Gothenburg, Sweden

**Keywords:** Guillain–Barré syndrome, Biomarkers, Neurofilament light chain, Albumin ratio, Prognosis, Diagnosis

## Abstract

**Background:**

Elevated neurofilament light chain (NfL) levels are associated with worse prognosis in Guillain–Barré syndrome (GBS). Our objectives were to determine the utility of serum NfL (sNfL), cerebrospinal fluid (CSF)/serum NfL ratio and NfL index as prognostic and diagnostic biomarkers for GBS.

**Methods:**

We measured NfL in serum and/or CSF obtained from 96 GBS patients between 1989 and 2014 in western Sweden. The sNfL *Z*-scores, NfL ratios and NfL indices were calculated. Outcome was determined with the GBS disability scale (GBSDS) at 3 and 12 months. NfL parameters in GBS were compared with healthy controls (HC), multiple sclerosis (MS), and amyotrophic lateral sclerosis (ALS).

**Results:**

The sNfL *Z*-score was higher for GBSDS > 2 at 3 months (median [IQR], 3.5 ng/L [3.2–4.0], vs 2.6 [1.7–3.4], *p* = 0.008) and at 12 months (3.6 ng/L [3.5–3.8] vs 2.6 [1.8–3.5], *p* = 0.049). NfL ratio and index were not associated with outcome. The area under the curve (AUC) for sNfL *Z*-score was 0.76 (95% CI 0.58–0.93, *p* < 0.0001) for GBSDS > 2 at 3 months. NfL ratio and index were lower in GBS than HC, MS, and ALS. The AUC for the NfL ratio was 0.66 (95% CI 0.55–0.78, *p* = 0.0018) and for the NfL index 0.86 (95% CI 0.78–0.93, *p* < 0.0001).

**Discussion:**

Our results confirm sNfL as prognostic biomarker for GBS and the precision was improved using the age-adjusted sNfL Z score. NfL index and Qalb are potential diagnostic biomarkers for GBS.

**Supplementary Information:**

The online version contains supplementary material available at 10.1007/s00415-024-12679-5.

## Introduction

Guillain–Barré syndrome (GBS) is a subacute inflammatory polyradiculoneuropathy involving intrathecally located nerve roots and peripheral nerves. Although GBS is usually a monophasic disease with good recovery, approximately 20% cannot walk independently at 12 months, and there is a 3–7% mortality [1]. Several clinical, demographic, and electrophysiological factors are associated with poorer prognosis in GBS [2, 3].

The pathophysiology of GBS is heterogeneous and can be classified based on electrophysiological investigations as demyelinating or axonal, depending on the primary target of injury. The axonal subtype is associated with a higher risk of long-term disability as axonal regeneration is slow and often incomplete compared with remyelination [4, 5].

During axonal damage or degeneration, the subunit neurofilament light chain (NfL) is released and measurable in cerebrospinal fluid (CSF) and blood. NfL has been established as a diagnostic and prognostic biomarker and may reflect disease progression in several neurological diseases, including GBS [6–10]. GBS patients with high concentrations of NfL in serum (sNfL) at clinical onset are more likely to be admitted to the intensive care unit (ICU), have more prolonged hospital admissions, are more disabled at discharge [6], and appear to have an increased risk of severe irreversible disability [7].

NfL concentrations in CSF and serum are non-linearly associated with age and body mass index (BMI) [11, 12]. Thus, the utility of fixed cut-off values for sNfL in individual assessments is limited in diseases that affect patients of all ages and different BMIs. Using a *Z*-score for sNfL levels, these confounding factors are taken into account, and the *Z*-score has been validated for patients with multiple sclerosis (MS) and found to predict disease progression [13].

Because GBS often affects both an intrathecal and an extrathecal part of the nerve, released NfL from nerve roots and the peripheral nerves may both contribute to increased blood NfL levels. There is no structural difference between peripherally or intrathecally synthesized NfL. However, the origin of NfL in GBS might be determined by calculating the CSF/serum NfL ratio or the NfL index (CSF/serum NfL ratio divided by the CSF/serum albumin ratio [Qalb]), which also accounts for the integrity of the blood-cerebrospinal fluid barrier (BCSFB). Lower NfL ratio or NfL index has been found in patients with axonal/mixed subtype of GBS compared with demyelinating GBS, indicating proportionally greater amounts of peripherally derived NfL in this subtype of GBS [14].

In our study, we aimed to further explore the clinical utility of NfL as a biomarker in GBS. We investigated the potential of sNfL *Z*-score, NfL ratio, NfL index, and Qalb as biomarkers of clinical outcome and for the classification of subtypes in a large cohort of GBS patients. In addition, we compared the NfL ratio and NfL index of GBS patients with those of three control populations: healthy controls (HC), patients with MS, and patients with amyotrophic lateral sclerosis (ALS). As MS is primarily a CNS (central nervous system) disease and ALS usually involves motorneurons in both the CNS and the PNS (peripheral nervous system), but in contrast to GBS, has essentially preserved integrity of the BCSFB, we hypothesized that by calculating the NfL ratio and NfL index in these study cohorts, we could determine the source of NfL and the effect of a damaged BCSFB on NfL levels. Based on these results, we evaluated NfL ratio and NfL index as diagnostic biomarkers.

## Methods

### Study population and clinical assessments

Patients with suspected GBS were prospectively evaluated for inclusion in the study between 1989 and 2014 at the Department of Neurology, Sahlgrenska University Hospital, Gothenburg, Sweden. Patients who fulfilled the Brighton diagnostic criteria for GBS[15] were included, however those with neurological comorbidities were excluded. Based on information from medical records, the GBS disability scale (GBSDS) was used to assess disability retrospectively [16]. Outcome was dichotomized into those with the ability and those unable to walk unsupported (GBSDS > 2) at three and 12 months and the need for respiratory support. Clinical subtype was classified according to the Wakerly criteria as classic, Miller Fisher syndrome (MFS), pharyngeal–brachial–cervical variant (PCB), paraparetic or bifacial weakness with distal paraesthesias (BWDP) [17]. Electrophysiological subtype was classified as normal, acute inflammatory demyelinating polyneuropathy (AIDP), acute motor/sensorimotor axonal neuropathy (AMAN/AMSAN), or equivocal based on results from nerve conduction studies (NCS), performed at the Neurophysiological Laboratory, Sahlgrenska University Hospital, and interpreted by certified neurophysiologists. Information on preceding infections and treatment was collected from medical records.

### Control populations

Three control populations were retrieved from the Department of Neurology at Sahlgrenska University Hospital, and their demographics and clinical characteristics are shown in Table 1.

**Table 1:** Baseline demographics and clinical characteristics

	HC (*n* = 73)	GBS (*n* = 96)	MS active (*n* = 24)	MS non-active (*n* = 39)	ALS (*n* = 34)
Age Mean ± SD	52.8 ± 23.3	51.8 ± 16.4	35.3 ± 10.3	37.6 ± 12.3	65.9 ± 11.3
Sex Male %	58.9	63.9	25.0	33.3	52.9
Disease duration at sampling Median (IQR)	NA	9.0 (5.0–17.5) days	7 (3.3–11.5) years	7.0 (4.0–13.0) years	1.9 (0.6–3.3) years
GBSDS at the time of first sample Median (IQR)	NA	3 (2–4)	NA	NA	NA
EDSS at the time of sample Median (IQR)	NA	NA	2.5 (1.5–4.0)	3.0 (2.0–4.0)	NA

*HC* healthy controls, *GBS* Guillain-Barré syndrome, *MS* Multiple Sclerosis, *ALS* Amyotrophic Lateral Sclerosis, *LP* lumbar puncture, *GBSDS* Guillain–Barré disability scale, *EDSS* expanded disability status scale, *NA* not available

#### Healthy controls

The cohort consisted of 73 neurologically healthy individuals who consented to donate serum and CSF samples to a research biobank while undergoing spinal anesthesia for an elective orthopedic procedure.

#### Amyotrophic lateral sclerosis

The cohort consisted of 34 patients with amyotrophic lateral sclerosis (ALS), all fulfilling the El Escorial diagnostic criteria for ALS [18].

#### Multiple sclerosis

From a previously published cohort of relapsing–remitting multiple sclerosis (RRMS) [19], 24 patients with ongoing disease activity and 39 patients without activity were used for comparison. All patients fulfilled the 2017 revised McDonald diagnostic criteria for MS[20]. Disease activity was defined as clinical relapse (symptom lasting > 24 h that could not be explained by any other reason) and/or one or more gadolinium-enhancing lesions on magnetic resonance imaging (MRI).

### Sample collection

Serum and CSF were collected from GBS patients during hospital admission and/or follow-up. Only NfL and albumin concentrations determined in samples collected within 30 days from the GBS onset were included in the statistical analyses. In the ALS cohort, serum and CSF samples were obtained between 2014 and 2016 during the diagnostic workup (*n* = 19) or later in the disease course (*n* = 25). Serum and CSF samples were obtained from MS patients during different stages of activity and disease course between 2006 and 2014. In patients with active disease, samples were obtained within three months from the onset of clinical or radiological activity. All samples were stored at – 80 °C until biomarker analysis.

### Analysis of NfL

All biomarker analyses were performed by board-certified laboratory technicians blinded to clinical data. To minimize variation, baseline and follow-up samples for the GBS cohort were analyzed side by side on each assay plate using one batch of reagents. In addition, samples from healthy controls were randomly analyzed in each assay plate. Analysis of the MS and ALS cohort were performed separately. All analyses were performed at room temperature.

The analyses of serum and CSF NfL were performed using the Simoa^®^ NEUROLOGY NF- light Advantage Kit (Quanterix, Billerica, MA). Briefly, the samples, QCs (quality control samples) and calibrator stock were removed from storage and allowed to thaw at room temperature. The RGP reagent was shaken for 30 min at 800 rpm and heated to 30 °C. The calibrators, samples and QCs were vortexed for 30 s at 2000 rpm. For serum NfL, the internal calibrators, plasma samples, and QCs were additionally centrifuged for 10 min at 4000 g. CSF samples were diluted 40 × with sample dilution reagent. Calibrators, samples and QCs were added to the plate and covered with sealing tape. Reagents, samples and calibrators were run in the HD-X Analyzer using a 4 × dilution for the plasma samples and 1 × dilution for the CSF samples.

### Analysis of albumin

Albumin levels were measured by immunonephelometry on a Beckman Immage Immunochemistry system (Beckman Instruments, Beckman Coulter, Brea, CA, USA).

### Calculations of the Qalb, NfL ratio, NfL index and sNfL *Z*-scores

The Qalb was calculated by dividing CSF albumin by serum albumin [21]. The NfL ratio was calculated by dividing CSF NfL by sNfL. The NfL index was calculated by dividing the NfL ratio by the Qalb [14].

sNfL *Z*-scores were calculated based on age and sNfL values using the web-based Serum Neurofilament light Chain Reference App [22]. As we did not have information on BMI for our cohorts, calculations were done assuming a BMI of 25 for all subjects.

### Statistical analysis

Statistical analysis was performed using IBM SPSS Statistics (version 29.0.2.0) and GraphPad Prism 10 (version 10.2.2).

Descriptive statistics for continuous variables are presented as mean and standard deviation if normally distributed, median and interquartile range if not. Categorical variables are expressed as counts. Since NfL is non-normally distributed, values are expressed as median and interquartile range. Non-parametric tests, Mann–Whitney *U* test and Kruskal–Wallis, were used to compare groups. Wilcoxon matched-pair signed rank test was used to compare paired samples. Dunn’s test was used for multiple comparisons. Multiple logistic regression analysis was used to adjust for confounding by age. Spearman Rank correlation coefficient was used to assess the correlation between sNfL and Qalb. A *p* value of < 0.05 was considered significant. Receiver operator characteristic (ROC) curves were calculated assuming non-parametric distribution. Sensitivity and specificity were calculated using Youden´s index.

### Ethical considerations

Approval was obtained from the Swedish Ethical Review Authority separately for each of the cohorts. Approval numbers are EPN 650–16 (the GBS cohort), EPN 460–13 (the HC cohort), EPN 298–14 (the ALS cohort), and EPN 2005:253 (the MS cohort).

## Results

### Clinical characteristics of the GBS cohort

Baseline demographics and clinical characteristics are presented in Table 1. Ninety-six GBS patients, 34 women, were included in the study. The clinical GBS subtypes according to the Wakerly criteria were classic (*n* = 80), paraparetic (*n* = 3), PCB variant (*n* = 3), BWDP (*n* = 1) and MFS (*n* = 9). According to the neurophysiological examination, cases were classified as AIDP (*n* = 46), AMAN/AMSAN (*n* = 10), equivocal (*n* = 9), and normal (*n* = 18). NCS was missing for 11 patients. The albumin ratio of GBS subjects was elevated (*n* = 70), normal (*n* = 26), and not done (*n* = 1). Preceding infection was reported for 75 patients with respiratory tract infections (*n* = 49), gastrointestinal tract infections (*n* = 13), and others (*n* = 2). Treatment consisted of a five days course of intravenous immunoglobulin (IVIG 0.4 g/L/day, *n* = 42), plasmapheresis (*n* = 24), a second or more IVIG five days course (*n* = 7), or IVIG and plasmapheresis in combination (*n* = 3). Nine patients did not receive treatment, and information was missing for 11. The median GBSDS was 2 (IQR 2–4) at diagnosis, 4 at nadir (IQR 2–4), and 1 (IQR 0–2) at the last follow-up 1 (median 340 days, IQR 88–738).

Eighty-nine GBS patients had serum (*n* = 21), CSF (*n* = 26), or both (*n* = 42) collected within 30 days from clinical onset. Additionally, eight patients had only samples collected later in the disease course: serum (*n* = 6), CSF (*n* = 1), or both (*n* = 1). Serial serum samples (3–11 samples) were available for 19 patients, and CSF (2–3 samples) were obtained from 18 patients.

### Comparison between GBS patients and healthy controls

GBS patients had higher sNfL levels than HC (median [IQR] 50.2 ng/L [16.7–209 ng/L] vs. 12.3 ng/L [6.1–19.8 ng/L], age-adjusted *p* < 0.0001) and CSF NfL levels (972 ng/L [510–972 ng/L] vs 499 ng/L [264–983 ng/L], age-adjusted *p* < 0.0001) compared with HC. The sNfL *Z*-scores were significantly higher in GBS patients (3.1 [1.9–3.7]) compared with HC (0.7 [− 0.2 to 1.5], *p* < 0.0001), and the NfL ratio was significantly lower (31 [14.1–68] vs 42.4 [33.3–55.5], *p* = 0.02).

### The association between NfLvalues, baseline clinical characteristics and GBS severity

**Fig. 1:**
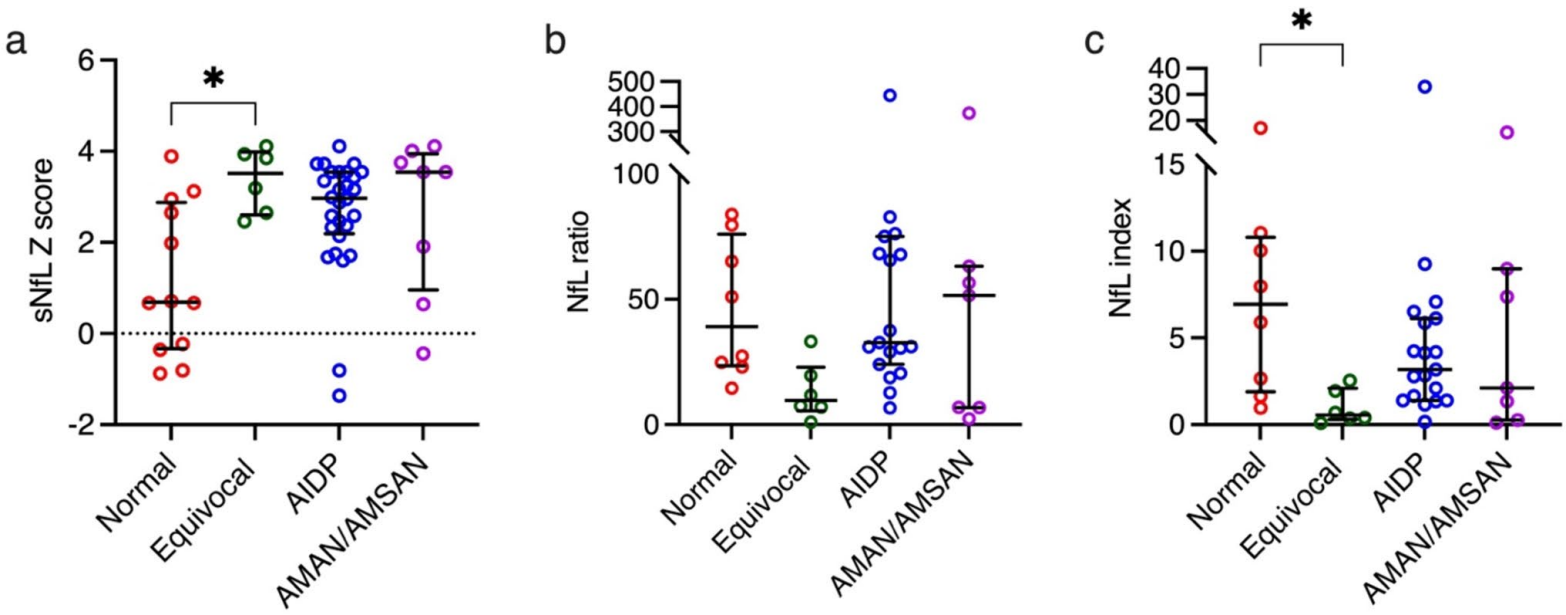
sNfL *Z*-score (**a**), NfL ratio (**b**), and NfL index (**c**) in different neurophysiologocial subtypes. Line and whiskers represent median and interquartile range, dots individual values. *p < 0.05, stastitically significant results from multiple comparisons analyses are shown. Abbreviations: *NfL* Neurofilament light chain, *AIDP* acute inflammatory demyelinating polyneuropathy, *AMAN/AMSAN* acute motor/sensorimotor axonal neuropathy

The association between sNfL levels, sNfL *Z*-score, NfL ratio, NfL index, Qalb and demographics, clinical characteristics, and GBS severity was evaluated (supplementary data: Table 1). NfL levels in serum and CSF and sNfL *Z*-scores increased with increasing GBSDS at diagnosis, *p* = 0.004, < 0.0001 and 0.0002 respectively. Patients with AMAN/AMSAN had higher sNfL (Med [IQR] 234 ng/L [9.4–341 ng/L]) and sNfL *Z*-scores (3.5 [1.0–3.9]) than those with normal neurophysiological subtype (sNfL 6.9 ng/L [5.3–20.4 ng/L], sNfL *Z*-score 0.69 [− 0.3 to 2.9]) or AIDP (sNfL 37.3 [23.4–111], sNfL *Z*-score 3.0 [2.2–3.5]), *p* = 0.002 and 0.02 respectively (Fig. 1). Patients with MFS had higher sNfL levels (187 ng/L [51.2–563 ng/L]) and sNfL *Z*-scores (3.7 [3.2–3.9]) than patients with classic GBS (sNfL 40.5 ng/L [18.8–197 ng/L], sNfL *Z*-score 3.0 [2.1–3.6]), PCB (6.0 ng/L [5.2–6.9], sNfL *Z*-score − 0.23 [− 0.2 to 0.7]) and paraparetic subtype (sNfL 66.6 ng/L [4.2–129 ng/L], sNfL *Z*-score 1.4 [− 0.36 to 3.2]), *p* = 0.03 and 0.002 respectively. The NfL index was higher in patients with normal neurophysiological subtype (6.9 [1.9–10.8]) compared with patients with AIDP (3.2 [1.4–6.1]), AMAN/AMSAN (2.1 [0.3–9.0]) and equivocal (0.6 [0.3–2.1]), *p* = 0.04. Multiple comparisons of sNfL and sNfL *Z*-score in clinical and neurophysiological suptypes are shown in supplementary data, Table 2–5. NfL ratio, NfL index and Qalb at baseline was otherwise not significantly influenced by gender, GBSDS, clinical GBS subtype, type of preceding infection, or neurophysiological subtype.

### Evolvement of NfL levels over time

To map the evolution of NfL levels over time, we calculated the median values and IQR of sNfL, CSF NfL and the NfL ratio depending on which week after clinical onset the samples were collected and analyzed sNfL and CSF NfL in available serial samples.

Between baseline and week two, the median sNfL level increased from 16.5 ng/L (IQR 5.5–46.2 ng/L) to 89.5 ng/L (IQR 18.3–291 ng/L), *p* = 0.003. The increase in CSF NfL was more modest, median 617 ng/L (IQR 389–1058 ng/L) to median 828 ng/L (IQR 340–3808 ng/L, *p* = 0.27). While the sNfL concentration peaked in week five, the CSF NfL level peaked in week four.

**Fig. 2:**
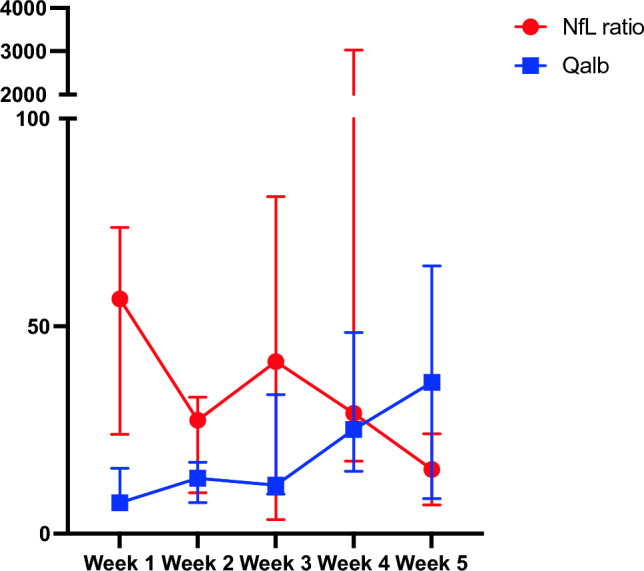
Evolvement of NfL ratio and Qalb from week one to week 5 after symptom onset expressed as median and interquartile range. *NfL* neurofilament light chain; *Qalb* albumin quotient

The median NfL ratio decreased in the first two weeks, from 56.6 (IQR 23.9–73.7) to 32.9 (9.9–27.3), *p* = 0.09, while the Qalb rose from 7.5 (IQR 6.4–15.8) to 13.4 (IQR 17.3–7.6), *p* = 0.23 (Fig. 2).

**Fig. 3:**
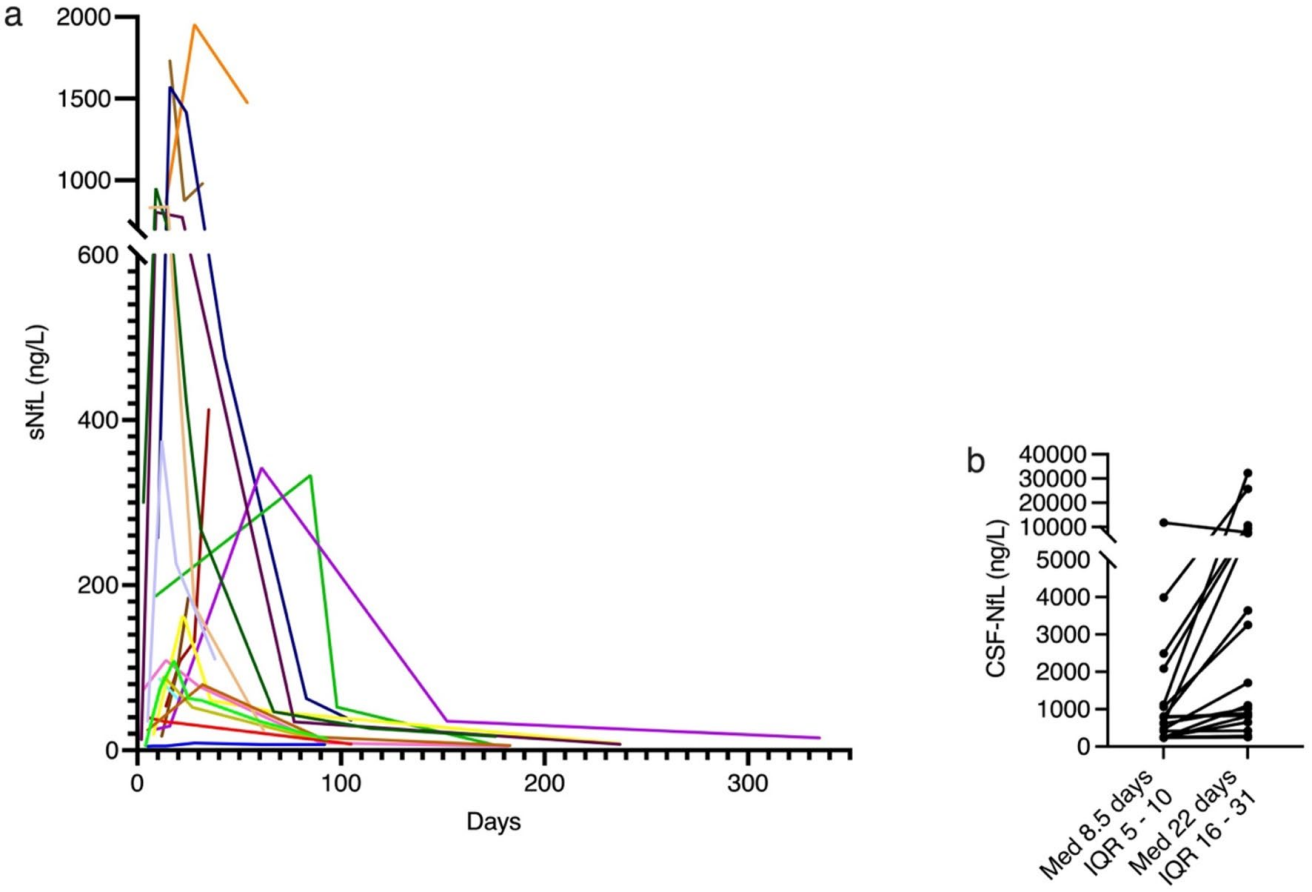
**a** Serial sNfL levels in individual patients by days from clinical GBS onset. **b** CSF NfL levels from clinical onset (week one) and at follow-up (week three). Abbreviations: sNfL serum neurofilament light chain and *CSF-NfL* cerebrospinal fluid neurofilament light chain, *Med* median, *IQR* interquartile range

Nineteen patients had three or more serum samples collected between day 3 and 335 from symptom onset (mean days 49) and the average duration from clinical onset to sNfL peak levels was 22.8 days (Fig. 3a).

Two CSF samples were available from 18 patients. The average duration between samples was 20.8 days (range 3–174). Median CSF-NfL increased from 709.5 ng/L (IQR 254–1362 ng/L) to 1402 ng/L (IQR 774–8170 ng/L) (*p* < 0.001) (Fig. 3b).

### NfL and GBSDS outcome

We dichotomized patients into severe GBS (GBSDS > 2, i.e., inability to walk) and those with normal or less disability at three and 12 months. Patients with MFS and PBC variants were excluded from this analysis, except for the need of respiratory support, since the ability to walk is usually not impaired in these subtypes. Results are shown in Table 2. NfL levels in serum and CSF and the sNfL *Z*-scores were significantly higher in those with severe GBS and those requiring respiratory support compared with less disabled GBS patients. No association was shown between the NfL ratio/NfL index and GBS severity. The Qalb was significantly higher in those needing respiratory support and with GBSDS > 2 at three months but not at 12 months.

**Table 2:** NfL parameters and albumin quotient compared between groups based on outcome

	sNfL < 30 d ng/L Med (IQR)	*P* value	CSF-NfL < 30 d ng/L Med (IQR)	*P* value	sNfL *Z*-score Med (IQR)	*P* value	NfL ratio Med (IQR)	*P* value	NfL index Med (IQR)	*P* value	Qalb	*P* value
Respiratory assistance												
Yes	*n* = 14 197 (64–399)	*0.02*^*a*^	*n* = 15 3770 (1303–16718)	*0.013*^*b*^	*n* = 14 3.7 (3.2–4.0)	*0.004*	*n* = 8 25 (7–67)	*0.73*	*n* = 8 1.4 (0.2–4.2)	*0.35*	*n* = 8 22 (14–45)	*0.012*
No	*n* = 49 32.8 (12.4–121)		*n* = 51 714 (444–1511)		*n* = 49 2.7 (1.6–3.5)		*n* = 25 29 (17–61)		*n* = 25 2.6 (1.3–6)		*n* = 25 11 (7.6–17)	
GBSD > 2 at 3 months												
Yes	*n* = 11 194 (33–554)	*0.01*^*a*^	*n* = 11 2620 (1303–74753)	*0.008*^*b*^	*n* = 11 3.5 (3.2–4.0)	*0.008*	*n* = 7 30 (6.7–68)	*0.69*	*n* = 7 1.4 (0.1–4.2)	*0.24*	*n* = 7 24.3 (11–47)	*0.026*
No	*n* = 38 31 (15–100)		*n* = 40 812 (464–1501)		*n* = 38 2.6 (1.7–3.4)		*n* = 24 30 (20–63)		*n* = 24 2.4 (1.4–6.1)		*n* = 24 12.3 (7.6–17)	
GBSD > 2 at 12 months												
Yes	*n* = 4 197 (184–511)	*0.02*^*a*^	*n* = 5 74,753 (1961–185,842)	*0.006*^*b*^	*n* = 4 3.6 (3.5–3.8)	*0.049*	*n* = 3 6.7 (0.96–374)	*0.42*	*n* = 3 0.2 (0.1–15)	*0.42*	*n* = 3 24 (10–25)	*0.28*
No	*n* = 45 33 (16.5–112)		*n* = 46 828 (504–2122)		*n* = 45 2.6 (1.8–3.5)		*n* = 28 30.8 (19.8–65)		*n* = 28 2.4 (1.4–5.9)		*n* = 28 13 (7.9–19)	

Abbreviations: *sNfL* serum neurofilament light chain, *CSF* cerebrospinal fluid, *Qalb* albumin quotient, *GBSDS* Guillain – Barré disability scale, *Med* median, *IQR* interquartile range

^a^ age-adjusted p value by multiple logistic regression with log sNfL and agegrou*p* < 65 years or ≥ 65 years as variables

^b^ age-adjusted p value by multiple logistic regression with log CSF NfL and agegrou*p* < 65 years or ≥ 65 years as variables

### sNfL *Z*-score to determine GBS prognosis

**Fig. 4:**
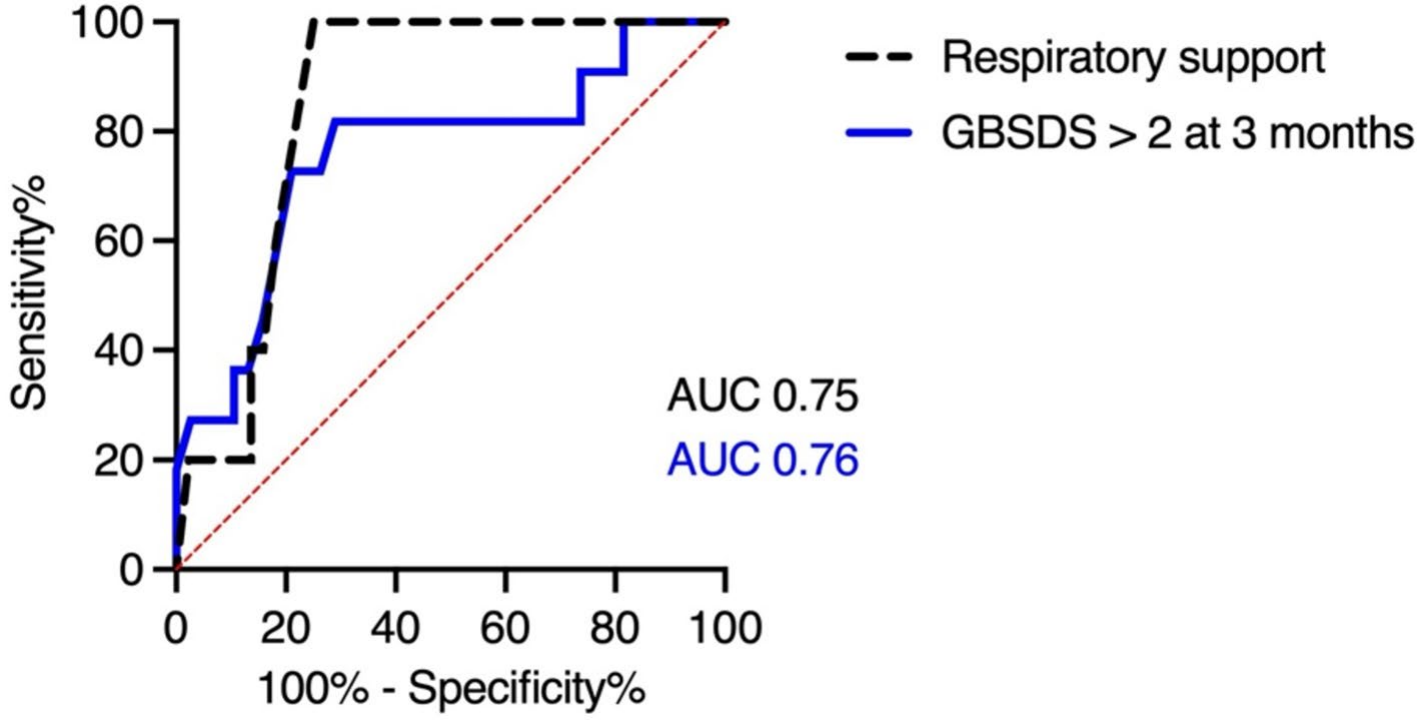
ROC curves of the sNfL *Z*-score for respiratory support and GBSDS > 2 at three months. Abbreviations: *GBSDS* Guillain–Barré syndrome disability sc*al*e*, AUC* area under the curve

We evaluated the potential of the sNfL *Z*-score to predict the risk of severe residual disability in GBS. ROC analysis was carried out for respiratory support and GBSDS > 2 at three months (Fig. 4). A *Z*-score of > 3.2 had a sensitivity of 82% and specificity of 71% for GBSDS > 2 at three months and a sensitivity of 79%, and a specificity of 65% for requiring respiratory support. Given that 25% of patients will need respiratory support, a sNfL Z-score of > 3.2 will have a positive predictive value (PPV) of 43% and a negative predictive value (NPV) of 91%.

### Comparison of albumin ratio, Nfl ratio and NfL index as diagnostic biomarkers for GBS

We excluded MFS and PCB subtypes from the comparison analysis as these rare GBS subtypes usually have low impact on peripheral nerves compared with classic GBS and therefore may be considered outliers among GBS subtypes.

The Qalb and the sNfL concentration correlated significantly in GBS patients (*r* = 0.4, *p* = 0.02). No correlation was found between Qalb and sNfL in the MS or ALS populations. Qalb was not available for HCs (Supplementary data, Table 6).

**Fig. 5:**
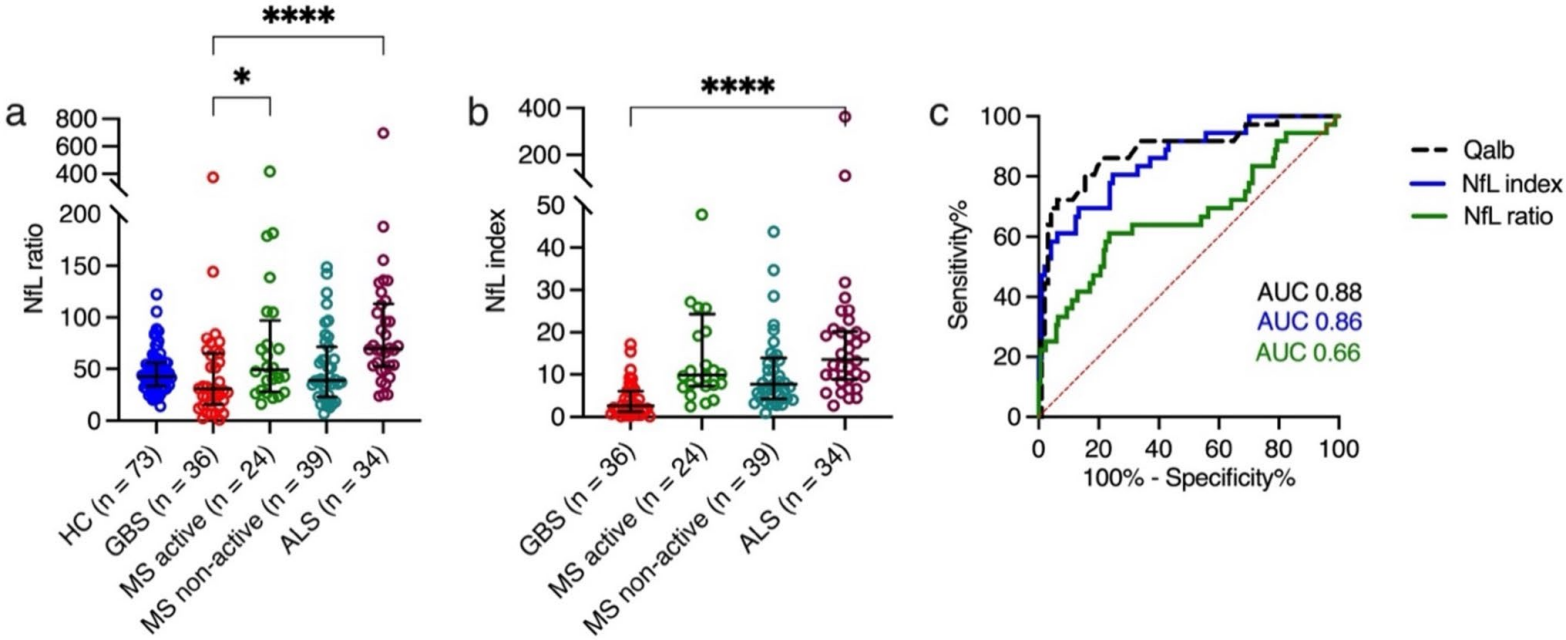
**a, b** Comparison of NfL ratio and NfL index between study populations and **c** ROC curves for NfL ratio, NfL index and Qalb**.** Dots represent individual values, line and whiskers median and interquartile range. **a** NfL ratio in HC, GBS, MS and ALS. **b** NfL index in GBS, MS an ALS. *p < 0.05, **** *p* < 0.0001. **c** ROC for NfL index and Qalb in GBS versus MS and ALS and for NfL ratio in GBS versus MS, ALS and HC. *NfL* neurofilament light chain*, Qalb* albumin quotient*, HC* healthy controls*, GBS* Guillain–Barré syndrome*, MS* multiple sclerosis*, ALS* amyotrophic lateral sclerosis, *ROC* receiver operator charachteristics curve

The NfL ratio was lower in GBS patients (median [IQR] 30.8 [15.6–65]) compared with HC (42.4 [ 33.3–55.5]), active MS (49.2 [ 27.9–96.9]), non-active MS (38.9 [23.1–71.3]) and ALS (69.4 [52.2–113]), *p* < 0.0001 (Fig. 5a). After applying multiple comparison tests, the difference between NfL ratios was statistically significant between GBS and active MS (*p* = 0.048) and between GBS and ALS (*p* < 0.0001) (Supplementary data, Tables 7 and 8).

To estimate the influence of an impaired BCSFB on the sNfL concentration, we calculated the NfL index. The NfL index was statistically significantly lower in GBS (median [IQR] 2.6 [1.2–6.1]) compared with active MS (9.8 [7.3–24.3]) non-active MS (7.7 [4.3–13.9]), and ALS (13.6 [8.8–20.2]), *p* < 0.0001 (Fig. 5b). The multiple comparison test showed statistically significant differences between GBS and the other study populations (Supplementary data, Tables 7 and 9).

The Qalb, NfL ratio and NfL index were determined in GBS, MS and ALS patients and their diagnostic value for GBS was determined in ROC analyses.

The AUC for NfL ratio was 0.66 (95% CI 0.55–0.78, *p* = 0.0018), and at an NfL ratio < 37.5, the sensitivity was 64% (95% CI 48–75%) and the specificity 69% (95% CI 62–75%).

The ROC analysis for NfL index showed that the AUC was 0.86 (95% CI 0.78–0.93, *p* < 0.0001), and at an NfL index of < 6.9, the sensitivity was 81% (95% CI 65–90%) and the specificity 70% (95% CI 60–78%).

The AUC for Qalb was 0.88 (95% CI 0.81–0.95, *p* < 0.0001), and at an Qalb of > 7.1, the sensitivity was 83% (95% CI 68–92%) and the specificity 80% (95% CI 71–87%) (Fig. 5c).

## Discussion

Our results confirm that sNfL, a biomarker for neuroaxonal injury, reflects disease severity in GBS. We showed that the sNfL *Z*-score, which is easy to apply in clincal practice, appeared to be a promising tool for predicting GBS severity. However, the diagnostic value of Qalb seemed to be similar to that estimated for NfL index, indicating that the impaired BCSFB is a crucial diagnostic feature of GBS.

As previously reported, increased levels of NfL in serum and CSF are associated with short- and long-term outcomes in GBS [6, 7, 9, 23], but its age dependance limits its use in clinical practice. We showed that the *Z*-score of sNfL at clinical onset, which takes into account age, improved the precision for the predictive value of sNfL for assessing GBS severity. The NfL *Z*-score has previously been validated as a marker for disease activity in MS, but to our knowledge, it has not previously been applied to a cohort of GBS patients [13].

The modified Erasmus GBS outcome score (mEGOS) is a prognostic model based on three clinical variables: MRC (medical research council) grade at week one, preceding diarrhea and age. The accuracy to which mEGOS discriminates patients to outcome is comparable to what we report for the NfL *Z*-score[24]. The advantage of the *Z*-score is that it is a purely objective measure and does not rely on subjective information from the patient.

Our mapping of the temporal profile of NfL shows that sNfL levels start rising in the second week after clinical onset, with a peak in week four or five. Previous longitudinal data on sNfL in GBS is limited. An estimated sNfL peak at day 16 after first assessment has been reported, followed by sNfL normalizations after one year, which is congruent with our findings [9, 25]. Our estimation of the NfL peak is compatible with the subacute monophasic nature of GBS, with axonal damage reaching its height within four weeks and then subsiding. NfL levels peaked earlier in CSF than in serum, suggesting that the immune attack of nerve roots is more extensive or precedes that of perpipheral nerves or that there is a delayed influx of NfL to the peripheral circulation from the CNS due to damage of the blood–brain barrier.

Since sNfL generally correlates strongly with CSF NfL [26], it has been assumed that NfL in blood originates from the CNS. In recent years, however, an increasing number of reports have emerged regarding NfL levels in blood as a marker of activity in diseases limited to the PNS [27–29]. Our findings suggest that damage to both intrathecal nerve roots and peripheral nerves are sources of NfL and contribute to sNfL levels in GBS. The proportionally faster rise of NfL in serum than CSF in the first two weeks might indicate that damage to peripheral nerves precedes those affecting nerve roots.

When comparing clinical subtypes, we find that MFS and PCB variants had higher NfL ratios and indices than the other variants. Agreeing with the clinical presentation, this indicates less prominent engagement of the peripheral nerves. We did not find any significant difference in NfL ratio or index between axonal and demyelinating subtypes as previously reported [14]. The timing of sampling might explain this discrepancy. In the previous study, the mean time from clinical onset to sampling was 3.7 days in patients with mainly demyelinating subtype. In contrast, the interval from GBS onset to sampling was 17 days in patients with axonal or mixed subtype.

The NfL ratio was significantly lower in GBS than in MS, ALS, and HC, supporting a peripheral sNfL source in GBS. Although ALS affects both the upper and lower motor neurons, it appears to have a more prominent CNS involvement, leading to higher NfL ratios.

Our results align with a previous study that reported a lower NfL ratio in GBS patients than HC [14]. In contrast, no difference in NfL ratio was observed in GBS patients compared with HC, chronic inflammatory neuropathy, and non-inflammatory polyneuropathy [30]. One possible explanation for this discrepancy is that the latter study included patients with MFS and MFS overlap syndrome.

While albumin concentration in CSF depends on the integrity of BCSFB [31], the role of BCSFB appears to be limited for the NfL ratio [32, 33]. However, we observed a moderate correlation between Qalb and sNfL in our GBS cohort but not in MS or ALS. This may imply that increased sNfL concentrations are a result of impaired BCSFB. Therefore, NfL from damaged intrathecal nerve roots may contribute to increased blood NfL concentration. However, the correlation between Qalb and sNfL in GBS may also be caused by increases in Qalb and sNfL coinciding after the autoimmune attack. Another explanation that has been proposed is that the elevated Qalb in GBS is caused by an increase in rostrocaudal albumin gradient due to CSF flow obstruction by swollen nerve roots and not simply a reflection of the BCSFB function [34]. In this case, Qalb would not be an appropriate marker of BCSFB function and, therefore, should not be used to correct for increased passage of NfL from the CNS/CSF compartment into the blood.

It is currently unknown how NfL transfers from the interstitial fluid (ISF) of the CNS to the blood. An investigation of the association between sNfL and BCSFB permeability after cranial radiotherapy did not show a correlation between sNfL and BCSFB opening in mice measured by the uptake of 14C-sucrose and no correlation between sNfL and Qalb in humans [32]. Furthermore, elevated Qalb due to BCSFB dysfunction in patients with a specific type of frontotemporal dementia (FTD-3) did not significantly affect sNfL levels [33]. Thus, these and our data support that the lower NfL ratio in GBS patients compared with that of other investigated neurological disorders is mainly caused by prominent damage to peripheral nerves and less from the contribution of NfL from damaged intrathecal nerve roots and disruption of BCSFB.

Our study has several limitations that could have influenced our results. The analyses of sNfL from GBS patients and control cohorts were not done simultaneously. However, the inter-asssay variability of the Simoa® immunoassay for NfL has been estimated at 6% [35, 36]. Furthermore, the samples were obtained at different time points < 30 days from clinical onset. As we showed that NfL peaked after four weeks, the sampling time could have affected the prognostic value of NfL. Both sNfL and CSF NfL depend on age, but only sNfL on BMI [11, 37, 38]. When estimating the sNfL *Z*-scores, we adjusted for age, but because we did not have data on BMI, we could not control for this confounding factor. This may lead to a lower NfL ratio in patients with a low BMI. In particular, this could have affected the NfL ratio in severely disabled patients with ALS. Additionally, we could not determine Qalb and NfL index in HC due to missing Qalb assays.

## Conclusion

We show that sNfL *Z*-score is a promising prognostic biomarker in GBS. However, our results need to be validated, including adjustments of the sNfL *Z*-score for BMI. We found no association between the NfL ratio or NfL index and residual GBS disability. The NfL parameters are dynamic in GBS, and the timing of sampling after clinical onset is, therefore, important for interpretation. Our results support that the increased levels of sNfL in GBS derive from both peripheral nerves and intrathecally located nerve roots with the main contribution from damaged peripheral nerves. We confirm that increased Qalb is a crucial feature for GBS and that Qalb and NfL index are potential diagnostic biomarker for GBS. Further studies are needed to assess their usefulness.

## Supplementary Information

Below is the link to the electronic supplementary material.

Supplementary file1 (DOCX 34 KB)
