# Supplementary material for: Neurofilament light chain as a diagnostic and prognostic biomarker in Guillain–Barré syndrome
Source: J Neurol. 2024 Sep 9;271(11):7282–93. doi: 10.1007/s00415-024-12679-5 (PMC11561089; doi:10.1007/s00415-024-12679-5)
Supplement: Supplementary file 1 — Supplementary file1 (DOCX 34 KB) [file 415_2024_12679_MOESM1_ESM.docx]

|  | n | sNfL ng/L  Median (IQR) |  | sNfL Z- score |  | n | CSF-Nfl ng/L  Median (IQR) |  | n | NfL ratio  Median (IQR) |  | NfL index  Median (IQR) |  | Qalb  Median (IQR) |  |
| --- | --- | --- | --- | --- | --- | --- | --- | --- | --- | --- | --- | --- | --- | --- | --- |
| Gender | | | | | | | | | | | | | | | |
| Male, n  Female, n | 47  16 | 40.6 (20.4 – 212)  48.5 (14.0 – 164) | *0.58* | 3.1 (2.0 – 3.8)  1.0 (3.0 – 3.5) | 0.51 | 41  25 | 961 (471 – 6572)  1084 (506 – 6572) | *0.78* | 29  13 | 32 (9.9 – 66.7)  24.9(16.6 – 68) | *0.99* | 2.1 (0.8 – 6.3)  2.8 (1.5 – 8.0) | *0.35* | 14.9 (7.4 – 24.7)  9.3 (6.7 – 12.7) | *0.05* |
| GBSDS at diagnosis | | | | | | | | | | | | | | |  |
| 1  2  3  4  5 | 15  23  5  18  1 | 16.6 (5.5 – 40.6)  27.9 (15 – 107)  81 (33.4 – 230)  199 (72.8 – 569)  323 ( - ) | *0.004* | 2.0 (-0.4 – 3.0)  2.5 (1.6 – 3.5)  3.4 (1.9 – 4.0)  3.7 (3.2 – 4.0)  4.0 (-) | *0.0002* | 12  22  7  24  1 | 755 (333 – 1287)  639 (398 – 890)  3301 (524 – 16718)  2399 (1048 – 8924)  2237 ( - ) | *< 0.0001* | 11  15  3  12  1 | 33.1 (23 – 75)  31.1 (24.1 – 64.6)  20.5 (12.7 – 56.6)  13.4 (6.8 – 79.1)  6.9 ( - ) | *0.55* | 3.2 (1.9 – 6.5)  2.8 (1.4 – 7.4)  1.7 (1.4 – 9.0)  1.4 (0.2 – 6.4)  0.1 ( - ) | *0.26* | 11.8 (6.5 – 15.6)  8.5 (7.0 – 23.3)  7.7 (6.3 – 15.2)  16.8 (12.1 – 36.7)  64.5 ( - ) | *0.61* |
| Clinical subtype | | | | | | | | | | | | | | | |
| Classic  MFS  PCB  Paraparetic  BWDP | 49  9  3  2  0 | 40.5 (18.8 – 197)  187 (51.2 – 563)  6.0 (5.2 – 6.9)  66.6 ( 4.2 - 129 )  - | *0.03* | 3.0 (2.1 – 3.6)  3.7 (3.2 – 3.9)  - 0.23 (-0.2 – 0.7)  1.4 ( -0.36 – 3.2)  - | *0.02* | 57  5  2  1  1 | 1037 (607 – 3639)  1503 (429 – 55265)  382 ( 350 - 414 )  1012 ( - )  324 ( - ) | *0.26* | 32  6  3  1  0 | 29.8 (15.6 – 65)  74 (7.7-1808)  51 (37.5 – 83.9)  11.7 ( - )  - | *0.46* | 2.0 (1.0-5.9)  4.6 (1.2 – 15.8)  8 (3.2 – 17.1)  2.5 ( - )  - | *0.26* | 14.2 (8.6 – 22.5)  9.1 (4.7 – 26.3)  6.4 ( 4.9 – 11.8)  4.6 ( - ) | *0.09* |
| Neurophysiological subtype | | | | | | | | | | | | | | |  |
| Normal  AIDP  AMAN/AMSAN  Equivocal | 9  27  8  6 | 6.9 (5.3 – 20.4)  37.3 (23.4 – 111)  234 (9.4 – 341)  371 (36.5 - 1164) | *0.002* | 0.69 (-0.3 – 2.9)  3.0 (2.2 – 3.5)  3.5 (1.0 – 3.9)  3.5 (2.6 – 4.0) | *0.02* | 14  24  8  8 | 612 (325 – 1133)  1221 (697 – 3099)  564 (434 – 1937)  2066 (692 - 5521) | *0.07* | 8  19  7  6 | 39.2 (23.5 – 76.0)  32.7 (24.1 – 75.1)  51.6 (6.8 – 63.2)  9.6 (5.5 – 22.9) | *0.07* | 6.9 (1.9 – 10.8)  3.2 (1.4 – 6.1)  2.1 (0.3 – 9.0)  0.6 (0.3 – 2.1) | *0.04* | 6.9 (5.3 – 13.5)  12.8 (10.7 – 16.0)  8.8 (6.3 – 46.7)  17.3 (8.4 – 22.0) | *0.13* |
| Preceding infection | | | | | | | | | | | | | | |  |
| Respiratory  Gastrointestinal | 31  9 | 40.5 (11.1 – 187)  86.8 (15.0 - 365) | *0.48* | 3.0 (0.7 – 3.7)  3.5 (1.8 – 3.8) | *0.63* | 36  10 | 663 (412 – 2217)  982 (629 – 86543) | *0.62* | 19  6 | 33.1 (8.0 – 65.6)  48.0 (20.3 – 201) | *0.44* | 2.0 (0.7 - 7.4)  2.4 (1.4 – 10.8) | *0.48* | 12.8 (7.0 – 17.0)  19.5 (7.8 – 29.9) | *0.51* |
| Treatment | | | | | | | | | | | | | | |  |
| None  IVIG  PE  IVIG > 1  IVIG + PE | 7  38  8  5  3 | 16.5 (6.0 – 76.6)  40.5 (22.6 – 218)  139 (16.6 – 448)  404 (218 – 563)  32.8 (28 – 81) | *0.01* | 2.0 (0.7 – 3.1)  3.0 (2.2 – 3.7)  3.5 (2.0 – 3.9)  3.9 (3.7 – 4.1)  3.2 (2.1 – 3.2) | *0.06* | 8  30  16  4  2 | 531 (274 – 2147)  1168 (584 – 2289)  739 (487 – 13732)  2769 (1372 - 188153)  2304 ( 837 - 3770 ) | *0.24* | 6  29  2  2  1 | 25.2 (12.7 – 59.2)  30.6 (9.9 – 66.7)  218.7 (63.2 – 374)  13.7 (6.9 – 20- 5)  30.9 ( - ) | *0.38* | 3.8 (0.8 – 10.3)  2.6 (1.3 – 6.8)  8.4 (1.4 – 15.4)  0.7 (0.1 – 1.4)  4.2 ( - ) | *0.53* | 10.7 (4.7 – 17.0)  11.0 (7.4 – 18.6)  35.5 (24.3 – 46.7)  39.9 (15.2 – 64.5)  7.4 ( - ) | *0.14* |

**Table 1** NfL parameters and albumin quotient (Qalb) and baseline data. Samples were taken < 30 days from symptom debut. Abbreviations: NfL neurofilament light chain, GBSDS Guillain-Barré disability scale, MFS Miller-Fisher syndrome, PCB Pharyngeal-cervical-brachial variant, AIDP acute inflammatory demyelinating polyneuropathy, AMAN/AMSAN acute motor/sensory axonal neuropathy, IVIG intravenous immunoglobulin, PE plasma exchange.

|  | Mean rank difference | Summary | Adjusted P-value |
| --- | --- | --- | --- |
| Classic vs. Paraparetic | 9.796 | ns | >0.99 |
| Classic vs. MFS | -8.982 | ns | >0.99 |
| Classic vs. PCB | 26.63 | ns | 0.09 |
| Paraparetic vs. MFS | -18.78 | ns | >0.99 |
| Paraparetic vs. PCB | 16.83 | ns | >0.99 |
| MFS vs. PCB | 35.61 | * | 0.02 |

**Table 2** Results from multiple comparison analysis of sNfL between different clinical subtypes. *p ≤ 0.05.

|  | Mean rank difference | Summary | Adjusted P-value |
| --- | --- | --- | --- |
| Classic vs. MFS | -11.13 | ns | 0.56 |
| Classic vs. PCB | 25.31 | ns | 0.12 |
| Classic vs. Paraparetic | 11.48 | ns | >0.99 |
| MFS vs. PCB | 36.44 | * | 0.02 |
| MFS vs. Paraparetic | 22.61 | ns | 0.69 |
| PCB vs. Paraparetic | -13.83 | ns | >0.99 |

**Table 3** Results from multiple comparison analysis of sNfL Z-score between different clinical subtypes. * p ≤ 0.05.

| Dunn's multiple comparisons test | Mean rank diff, | Summary | Adjusted P Value |
| --- | --- | --- | --- |
| Normal vs. AIDP | -15.00 | * | 0.045 |
| Normal vs. AMAN/AMSAN | -19.75 | * | 0.032 |
| Normal vs. Equivocal | -27.00 | ** | 0.003 |
| AIDP vs. AMAN/AMSAN | -4.750 | ns | >0.99 |
| AIDP vs. Equivocal | -12.00 | ns | 0.41 |
| AMAN/AMSAN vs. Equivocal | -7.250 | ns | >0.99 |

**Table 4** Results from multiple comparison analysis of sNfL between different neurophysiological subtypes. * p ≤ 0.05. ** p ≤ 0.01.

| Dunn's multiple comparisons test | Mean rank diff, | Summary | Adjusted P Value |
| --- | --- | --- | --- |
| Normal vs. AIDP | -11.68 | ns | 0.19 |
| Normal vs. AMAN/AMSAN | -16.00 | ns | 0.15 |
| Normal vs. Equivocal | -22.04 | * | 0.03 |
| AIDP vs. AMAN/AMSAN | -4.321 | ns | >0.99 |
| AIDP vs. Equivocal | -10.36 | ns | 0.57 |
| AMAN/AMSAN vs. Equivocal | -6.042 | ns | >0.99 |

**Table 5** Results from multiple comparison analysis of sNfL Z-score between different neurophysiological subtypes. * p ≤ 0.05.

|  | sNfL/Qalb,  r (95% CI) | P-value |
| --- | --- | --- |
| GBS  (n=36) | 0.4 (0.07 – 0.68) | *p = 0.02* |
| MS active  (n = 24) | - 0.34 (-0.66 – 0.09) | *p = 0.12* |
| MS non-active  (n = 39) | 0.24 (-0.1 – 0.52) | *p = 0.15* |
| ALS  (n=35) | 0.19 (-0.17 – 0.50) | *P = 0.28* |

**Table 6** Correlation between sNfL and Qalb. NfL neurofilament light chain, GBS Guillain-Barré syndrome, MS multiple sclerosis, ALS amyotrophic lateral sclerosis, HC healthy controls.

|  | HC  (n=73) | GBS  (n=36) | MS active (n=24) | MS non-active  (n= 39) | ALS  (n=34) |  |
| --- | --- | --- | --- | --- | --- | --- |
| CSF-NfL  Med (IQR) | 499 (264-983) | 1027 (509-1955) | 950 (551-1980) | 510 (331-700) | 5620 (2434- 9103) |  |
| sNfL  Med (IQR) | 12.3 (6.1-19.8) | 35(10.3-153) | 17.3 (11.4-36.1) | 13.4 (9.3-18.9) | 69.3 (46-127) |  |
| Qalb  Med (IQR) | NA | 12.3 (7.6-19.5) | 5.2 (3.9-6.7) | 5.3 (4.1-6.3) | 6.2 (6.2-7.3) |  |
| NfL ratio  Med (IQR) | 42.4 (33.3-55.5) | 30.8 (15.6-65) | 49.2 (27.9-96.9) | 38.9 (23.1-71.3) | 69.4 (52.2 –113) | *p < 0.0001* |
| NfL Index  Med (IQR) | NA | 2.6 (1.2 – 6.1) | 9.8 (7.3-24.3) | 7.7 (4.3-13.9) | 13.6 (8.8-20.2) | *p < 0.0001* |

**Table 7** Comparison of NfL levels in serum and CSF, Qalb, NfL ratio and NfL index between Guillain-Barré syndrome (GBS), healthy controls (HC), multiple sclerosis (MS) and amyotrophic lateral sclerosis (ALS). s*NfL* serum neurofilament light chain, *CSF-NFL* cerebrospinal fluid neurofilament light chain, *Med* median, *IQR* interquartile range, *NA* not available.

| Dunn's multiple comparisons test | Mean rank diff, | Summary | Adjusted P Value |
| --- | --- | --- | --- |
| GBS (n = 36) vs. HC (n = 73) | -23.22 | ns | 0.22 |
| GBS (n = 36) vs. MS active (n = 24) | -39.43 | * | 0.048 |
| GBS (n = 36) vs. MS non-active (n = 39) | -19.59 | ns | 0.62 |
| GBS (n = 36) vs. ALS (n = 34) | -70.84 | **** | <0.0001 |

**Table 8** Results from multiple comparison analysis of NfL ratio between GBS and Healthy controls (HC), active multiple Sclerosis (MS), non-active multiple sclerosis and amyotrophic lateral sclerosis (ALS). *p ≤0.05, **** p ≤ 0.0001.

| Dunn's multiple comparisons test | Mean rank diff, | Summary | Adjusted P Value |
| --- | --- | --- | --- |
| GBS (n = 36) vs. MS active (n = 24) | -50.4 | **** | <0.0001 |
| GBS (n = 36) vs. MS non-active (n = 39) | -36.3 | *** | 0.0001 |
| GBS (n = 36) vs. ALS (n = 34) | -58.3 | **** | <0.0001 |

**Table 9** Results from multiple comparison analysis of NfL index between GBS and active multiple Sclerosis (MS), non-active multiple sclerosis and amyotrophic lateral sclerosis (ALS). *** p ≤ 0.001, **** p ≤ 0.0001.
